# Dietary L-arginine intake and the incidence of coronary heart disease: Tehran lipid and glucose study

**DOI:** 10.1186/s12986-016-0084-z

**Published:** 2016-03-15

**Authors:** Zahra Bahadoran, Parvin Mirmiran, Zhaleh Tahmasebinejad, Fereidoun Azizi

**Affiliations:** Student Research Office, Nutrition and Endocrine Research Center, Research Institute for Endocrine Sciences, Shahid Beheshti University of Medical Sciences, Tehran, Iran; Nutrition and Endocrine Research Center, Research Institute for Endocrine Sciences, Shahid Beheshti University of Medical Sciences, Tehran, Iran; Endocrine Research Center, Research Institute for Endocrine Sciences, Shahid Beheshti University of Medical Sciences, Tehran, Iran; No. 24, Shahid-Erabi St., Yeman St., Velenjak, Tehran, Iran

**Keywords:** L-arginine, Coronary heart disease, Hypertension

## Abstract

**Background:**

We investigated the association of regular dietary intake of L-arginine and both the incidence of coronary heart disease (CHD) and changes of blood pressure.

**Methods:**

Eligible adults (*n* = 2284) who participated in the Tehran Lipid and Glucose Study were followed for a mean of 4.7 years. Dietary intake of L-arginine was assessed at baseline (2006–2008); biochemical variables were evaluated at baseline and the follow-up examination. Multivariate Cox proportional hazard regression models adjusted for potential confounders were used to estimate the risk of CHD across tertiles of L-arginine intake. Linear regression models were also used to indicate the association of L-arginine intake with changes of serum lipids and blood pressure during the follow-up.

**Results:**

Mean age of participants (42.8 % men) was 38.2 ± 13.4, at baseline. During a mean 4.7 ± 1.4 y of follow-up, 57 participants experienced CHD events. A significant negative association was observed between plant-derived L-arginine intake and changes of both systolic and diastolic blood pressure, whereas animal-derived L-arginine intake was related to increased levels of diastolic blood pressure (*P* < 0.01). Participants in the 2^nd^ tertile (1.45–1.78 g/d) had a significantly increased risk of CHD events compared to the participants in the 1^st^ tertile (<1.45 g/d) (HR = 1.90, 95 % CI = 1.03–3.58). The risk of CHD had a decreasing trend across increasing plant-derived L-arginine intake (HR = 1.0, HR = 0.91, 95 % CI = 0.51–1.62, HR = 0.72, 95 % CI = 0.39–1.32, P for trend = 0.03).

**Conclusion:**

Higher intake of plant derived L-arginine may have a protective effect whereas animal-derived L-arginine may be a risk factor for development of hypertension and CHD events.

## Background

L-Arginine, a conditionally essential amino acid, is the main substrate of nitric oxide synthase (NOS) family enzymes and is responsible for the production of the endothelium-derived relaxing factor nitric oxide (NO), which is involved in regulatory mechanisms of the cardiovascular system [1, 2]. The body sources of L-arginine include dietary protein, endogenous synthesis and proteins turnover; endogenous synthesis of L-arginine in healthy adults is sufficient, however in some states such human growth stages, catabolic stress, and dysfunction of the small intestine or kidney, L-arginine is classified as a conditional amino acid, because under such conditions the endogenous synthesis of L-arginine is not sufficient to meet the human body requirement fo L-arginine [3]. Additionally, L-arginine has a number of direct effects on endothelial functions via antioxidant activity, decrease blood viscosity, inhibition of angiotensin-converting enzyme, stimulation of fibrinolysis, and some hormones such as glucagon, prolactine and growth hormone [4]. It has been indicated that any defect in L-arginine metabolism such as increased levels of asymmetric dimethylarginine (ADMA), a competitive inhibitor of NO synthase, are related to endothelial dysfunction and increased risk of cardiovascular disease [5].

It has been proposed that dietary L-arginine intake may be involved in the development of coronary heart disease (CHD) via modification of NO homeostasis. Some previous animal studies and clinical trials however reported some favorable effects on cardiovascular risk factors following acute or long-term administration of L-arginine by several mechanisms, including endothelium dependent vasodilation and inhibition of platelet aggregation [6–9]. This issue remains a challenging debate due to some controversial findings [10, 11]; in CAD patients and postmenopausal women, oral administration of 9 g/d L-arginine had no significant effects on NO production, flow-mediated dilation of the brachial artery, cell adhesion molecules E-selectin, intercellular adhesion molecule-1, and vascular cell adhesion molecule-1 [12, 13].

Some short-term beneficial effects of high-doses L-arginine supplementation however have been reported in diseases states, including hypertension and cardiovascular disease [9], it is not clear whether L-arginine in usual amount of dietary intake could prevent cardiovascular disease events. Findings of two previous population-based studies did not support the cardiovascular protective effects of regular dietary intake of L-arginine [14, 15].

There is limited data regarding the association of dietary L-arginine and its dietary sources in relation to CHD, and to the best of our knowledge this issue has not yet been investigated in the framework of a population-based prospective examination; such studies could provide more valuable information regarding long-term effects of L-arginine intake on cardiovascular outcomes. Moreover, considering some evidence regarding better utilization of plant-derived L-arginine in the body [16], the sources of dietary L-arginine may impress its cardiovascular effects, an issue that should be investigated in human studies. In this study, we aimed to evaluate the association of total dietary L-arginine intake as well as animal- and plant-derived L-arginine with the incidence of CHD in a national representative population.

## Methods

### Study population

**Fig. 1:**
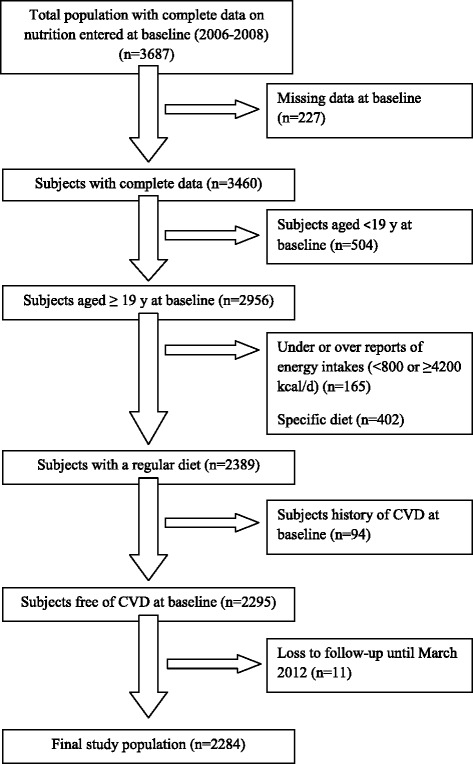
Diagram of the study population

This study was conducted within the framework of the Tehran Lipid and Glucose Study (TLGS), an ongoing community-based prospective study being conducted to investigate and prevent non-communicable diseases in a representative sample in the district 13 of Tehran, the capital city of Iran [17]. We recruited 2956 adult men and women (aged ≥ 19 y), with complete data (demographics, anthropometrics, biochemicals and dietary data), who participated in the third TLGS examination (2006–2008). Participants were excluded from the final analysis if they had unexplained energy intake (<800 kcal/d or > 4200 kcal/d) or were on specific diets (*n* = 567). Participants with cardiovascular disease history at baseline were also excluded (*n* = 94). The remaining participants (*n* = 2295) were followed until March 2012, with a mean period of 4.7 years from the baseline examination. Participants who had left the study (*n* = 11) were also excluded and final analyses was conducted on 2284 adults (977 men, 1307 women) (Fig. 1).

Written informed consents were obtained from all participants and the study protocol was approved by the Ethics Research Council of the Research Institute for Endocrine Sciences, Shahid Beheshti University of Medical Sciences.

### Demographic and anthropometric measures

Demographics, anthropometrics and biochemical measures were assessed both at baseline (2006–2008) and again at the follow-up examination (2010–2012). Trained interviewers collected information including demographic data, medical history, medication use and smoking habits, using pretested questionnaires. Weight was measured to the nearest 100 g using digital scales, while the subjects were minimally clothed, without shoes. Height was measured to the nearest 0.5 cm, in a standing position without shoes, using a tape meter. Body mass index was calculated as weight (kg) divided by square of the height (m^2^). Waist circumference was measured to the nearest 0.1 cm, midway between the lower border of the ribs and the iliac crest at the widest portion, over light clothing, using a soft measuring tape, without any pressure to the body. For blood pressure (BP) measurements, after a 15-minute rest in the sitting position, two measurements of BP were taken on the right arm, during a standardized mercury sphygmomanometer; the mean of the two measurements was considered as the participant’s BP.

### Biochemical measures

Fasting blood samples were taken after 12–14 h from all study participants at baseline and follow-up phase. Serum creatinine levels were assayed using kinetic colorimetric Jaffe method. Fasting serum glucose (FSG) was measured by the enzymatic colorimetric method using glucose oxidase. The standard 2 h serum glucose 2-h SG test was performed for all individuals who were not on anti-diabetic drugs. Serum total cholesterol (TC) was assayed using the enzymatic colorimetric method with cholesterol esterase and cholesterol oxidase. Triglyceride (TG) levels were measured by enzymatic colorimetric analysis with glycerol phosphate oxidase. High-density lipoprotein cholesterol (HDL-C) was measured after precipitation of the apolipoprotein B containing lipoproteins with phosphotungstic acid. The modified Friedewald formula was used to calculate LDL-C levels. Analyses were performed using Pars Azmoon kits (Pars Azmoon Inc., Tehran, Iran) and a Selectra 2 auto-analyzer (Vital Scientific, Spankeren, Netherlands). Both inter- and intra-assay coefficients of variation of all assays were < 5 %.

Serum creatinine levels were assayed using kinetic colorimetric Jaffe method. To calculate estimated glomerular filtration rate (eGFR), the CKD-EPI creatinine equation, developed by Chronic Kidney Disease Epidemiology Collaboration, was used. As a single equation CKD-EPI has been expressed as follows: eGFR = 141 × min (S_cr_/κ, 1)^α^ × max (S_cr_/κ, 1)^−1.209^ × 0.993^age^ × 1.018 [if female] × 1.159 [if black]. In this equation, S_cr_ is serum Cr in mg/dL; κ is 0.7 and 0.9 for men and women, respectively, α is −0.329 and −0.411 for men and women, respectively; min indicates the minimum of S_cr_/κ or 1, and max indicates maximum of S_cr_/κ or 1 [18].

### Dietary assessment

Dietary assessment was conducted at baseline. A validated 168-item food frequency questionnaire (FFQ) was used to assess typical food intake, and total L-arginine intake as well as L-arginine from animal and plant sources, over the previous year. L-arginine content of food items (mg/100 g of foods), were multiplied by the amount of daily intake of food items; dietary total intake of the participants was estimated by summing up of the obtained values. To stratify dietary L-arginine sources, amount of L-arginine intake was separately calculated by summing up of dietary L-arginine intake from plant sources (fruits, vegetables, nuts, legumes, grains) and animal sources (meats, processed meats, dairy, eggs).

The validity of the food frequency questionnaire has previously been evaluated by comparing food groups and nutrient values determined from the questionnaire with values estimated from the average of twelve 24-h dietary recall surveys [19]. The correlation coefficient for protein intake between the average of twelve 24-h recalls and the FFQ was 0.65 and 0.50 in men and women, respectively [19]. Trained dietitians with at least 5 years of experience in the TLGS survey, asked participants to designate their intake frequency for each food item consumed during the past year on a daily, weekly, or monthly basis. Portion sizes of consumed foods reported in household measures were then converted to grams [20]. However, since Iranian Food Composition Table is incomplete, and has limited data on nutrient content of raw foods and beverages, to analyze foods and beverages for their energy and nutrient content, we used the US Department of Agriculture Food Composition Table.

### Definition of terms

History of cardiovascular disease was defined as previous ischemic heart disease and/or cerebrovascular accidents. Family history of premature cardiovascular disease reflected any prior diagnosis of cardiovascular disease in first-degree female relatives aged below 65 years, or first-degree male relatives aged less than 55 years. Diabetes was defined as fasting serum glucose ≥ 126, 2 h serum glucose ≥ 200 or anti-diabetic medications [21]. Current smoker was defined as a person who smoked cigarettes daily or occasionally. Chronic kidney disease was defined as estimated eGFR < 60 mL/min per 1.73 m^2^ [22]. Diabetes was defined as fasting serum glucose ≥ 126, 2 h serum glucose ≥ 200 or anti-diabetic medications [21].

### Definition of outcome in our study

Details of the collection of cardiovascular outcome data have been described elsewhere [23]. Briefly, each participant was followed up for any medical event annually by phone calls. Information of any medical condition or event was collected by a trained nurse, a trained physician and by utilization of data from medical files. The collected data were evaluated and confirmed by an outcome committee consisting of an internist, an endocrinologist, a cardiologist, an epidemiologist and other experts. In the current study, event was considered as the first coronary heart disease (CHD) event, including definite myocardial infarction (MI) [with diagnostic electrocardiogram (ECG) and biomarkers], probable MI (positive ECG findings plus cardiac symptoms or signs plus missing biomarkers or positive ECG findings plus equivocal biomarkers), unstable angina (new cardiac symptoms or changing symptom patterns and positive ECG findings with normal biomarkers), angiographic proven CHD, and death from CHD [24].

### Statistical methods

Dietary intake of L-arginine was adjusted for total energy and protein intake, according to residuals methods [25]. The mean (SD) values and the frequency (%) of baseline characteristics of the participants with and without CHD event were compared using independent *t* test or chi square test, respectively. Dietary intakes of the participants across tertiles of dietary L-arginine intakes were compared using the analysis of variance (ANOVA).

Cox proportional hazard regression was used to assess the hazard ratios (HRs) of dietary L-arginine intakes for CHD. Time to event was defined by time of censoring or having event, whichever came first. We censored participants at the time of other causes of death, leaving the district or being in the study until March 2012 without event.

A univariate analysis was performed for potential confounding variables; variables with P_E_ < 0.2 in the univariate analyses were selected for the final multivariable models; P_E_ (P value for entry) determines which variables should be included in the multivariable model.

Linear associations of baseline L-arginine intakes with both changes of serum lipids and blood pressure during the follow-up period were estimated using linear regression models with adjustment of age, sex, energy and protein intakes.

We assessed dietary intakes of total L-arginine, plant-derived and animal-derived L-arginine as both continuous and categorical variables in the models. In the categorical model, amount of L-arginine and its subgroups was categorized into tertiles, given the 1st tertile as reference. In the continuous model, HR was calculated for each 1 SD increases in the intakes of L-arginine. Tree Cox proportional hazard regression models were defined; model 1 were adjusted for age (y) and sex (male/female); model 2 was further adjusted for BMI (kg/m^2^) and smoking status (yes/no); model 3 was additionally adjusted for hypertension (yes/no) and diabetes mellitus (yes/no). To assess the overall trends of HRs across tertiles of L-arginine intakes, the median of each tertile was used as a continuous variable in Cox proportional hazard regression models.

We set the statistical significance level at a two-tailed type I error of 0.05. All statistical analyses were performed using STATA version 12.0 (STATA, College Station, TX, USA).

## Results

Mean age of participants (42.8 % men) was 38.2 ± 13.4, at baseline. Mean dietary intakes of total L-arginine, plant-derived and animal-derived L-arginine was 4.0 ± 1.5, 2.2 ± 0.8 and 1.8 ± 0.9 g/d, respectively. Compared to controls, mean intakes of L-arginine was lower in subjects with CHD (3.8 ± 1.2 vs. 4.1 ± 1.5 g/d, *P* = 0.05).

During the average of 4.7 ± 1.4 y of follow-up, 57 participants experienced CHD event. The distributions of the major known CHD risk factors and some biochemical values for the participants who had CHD event and for those who did not are shown in Table 1. Risk factors for which there were significant differences between participants with and without CHD were age, smoking status, anthropometric measures, blood pressure, lipid profiles, and serum creatinine. Higher prevalence of diabetes (14.3 *vs.* 3.9, *P* = 0.003) and HTN (43.9 *vs.* 8.2, *P* = 0.001) was observed in subjects with CHD event compared to the rest of cohort. Dietary intakes of the participants across tertiles of total L-arginine are presented in Table 2. An increasing trend of dietary intakes of total energy, carbohydrate, cholesterol and protein was observed across increasing intakes of L-arginine (*P* = 0.001); compared to the lowest tertile, those in the highest tertile of L-arginine had also lower intakes of total fats as well as saturated and unsaturated fats (*P* = 0.001).

**Table 1:** Baseline characteristics of the participants

	Participants with CHD outcome *(n = 57)*	Participants without CHD outcome *(n = 2223)*	*P* value	Whole study population *(n = 2280)*
Age *(y)*	59.2 ± 9.7	37.6 ± 13.1	0.001	38.2 ± 13.4
Male *(%)*	59.6	42.3	0.007	42.8
Smoking *(%)*	22.8	11.5	0.003	11.7
Body mass index *(m* ^*2*^ */kg)*	28.4 ± 4.4	26.5 ± 4.8	0.004	26.5 ± 4.8
Waist circumference *(cm)*	97.2 ± 10.1	87.8 ± 13.3	0.001	88.0 ± 13.4
Serum creatinine *(μmol/L)*	99.5 ± 16.7	91.7 ± 13.5	0.001	91.9 ± 13.7
eGFR *(ml/min/1.73 m2)*	78.7 ± 13.0	79.8 ± 16.1	0.61	79.8 ± 16.1
Systolic blood pressure *(mm Hg)*	130 ± 17.4	109 ± 14.9	0.001	110 ± 15.3
Diastolic blood pressure *(mm Hg)*	80.0 ± 10.6	72.4 ± 10.3	0.001	72.6 ± 10.3
Fasting blood glucose *(mg/dL)*	106 ± 39.9	88.0 ± 16.6	0.001	88.9 ± 17.7
Total cholesterol *(mg/dL)*	216 ± 38.0	182 ± 36.8	0.001	183 ± 37.3
Serum triglycerides *(mg/dL)*	190 ± 107	132 ± 77.0	0.001	133 ± 79.0
LDL-C *(mg/dL)*	140 ± 31.2	112 ± 31.4	0.001	113 ± 31.6
HDL-C *(mg/dL)*	40.2 ± 8.0	43.3 ± 10.4	0.025	43.2 ± 10.4
TG/HDL-ratio	5.0 ± 3.1	3.4 ± 2.6	0.001	3.5 ± 2.6
Diabetes *(%)*	14.3	3.9	0.003	4.2
Hypertension *(%)*	43.9	8.2	0.001	9.1
Chronic kidney disease *(%)*	4.4	6.7	0.41	6.7

Data are mean ± SD unless stated otherwise (independent *t*-test for continuous variables and chi-square test for dichotomous variables was used

**Table 2:** Dietary intakes of the participants across tertiles of total L-arginine intakes

	Total L-arginine intakes			
	Tertile1	Tertile2	Tertile3	*P*
Dietary L-arginine *(g/d)*				
Range	<3.25	3.25–4.42	≥4.42	
Median	2.65	3.75	5.37	
L-arginine from animal sources *(g/d)*	1.09 ± 0.36	1.69 ± 0.44	2.67 ± 1.1	0.001
L-arginine from plant sources *(g/d)*	1.47 ± 0.39	2.10 ± 0.45	3.0 ± 0.83	0.001
Energy intake *(kcal/d)*	1587 ± 365	2217 ± 389	2976 ± 558	0.001
Carbohydrate *(% energy)*	56.3 ± 7.5	58.0 ± 7.2	57.4 ± 6.6	0.001
Protein *(% energy)*	11.8 ± 1.6	13.5 ± 1.5	15.6 ± 2.1	0.001
Total fats *(% energy)*	34.3 ± 7.6	30.9 ± 6.7	29.6 ± 5.7	0.001
Saturated fats *(% energy)*	11.2 ± 3.3	10.8 ± 7.3	10.1 ± 3.1	0.001
Monounsaturated fats *(% energy)*	12.0 ± 3.1	10.6 ± 2.7	10.2 ± 2.3	0.001
Polyunsaturated fats *(% energy)*	7.5 ± 2.6	6.3 ± 2.1	5.8 ± 1.8	0.001
Cholesterol *(mg/1000 kcal)*	85.0 ± 31.5	99.0 ± 41.0	118 ± 63.7	0.001
Total fiber *(g/1000 kcal)*	15.7 ± 7.3	16.4 ± 6.8	16.4 ± 5.8	0.06

Data are mean ± SD

Analysis of variance was used

Table 3 shows linear association of L-arginine intake at baseline with changes of serum lipids and blood pressure during the follow-up period. A significant negative association was observed between plant-derived L-arginine and changes of both systolic and diastolic blood pressure (β = -1.89, 95 % CI = −4.17, −0.03, and β = −2.78, 95 % CI = −4.61, −0.92). Animal-derived L-arginine intakes were related to increased levels of DBP (β = 1.80, 95 % CI = 0.14, 3.46); total L-arginine intakes were not significantly related with changes of serum lipids and blood pressure.

**Table 3:** Linear association (coefficient β and 95 % CI) of dietary intakes of L-arginine with changes of lipids and blood pressure during follow-up

	Total L-arginine intakes		
	Total L-arginine	Animal-derived L-arginine	Plant-derived L-arginine
Systolic blood pressure	−0.09 (−2.23, 2.05)	1.48 (−0.05, 3.51)	−1.89 (−4.17, −0.03)
Diastolic blood pressure	−0.55 (−2.31, 1.21)	1.80 (0.14, 3.46)	−2.78 (−4.61, −0.92)
Serum triglycerides	0.42 (−3.66, 4.50)	2.11 (−0.71, 5.95)	−2.11 (−6.32, 2.14)
HDL-C	1.62 (−0.08, 3.33)	1.75 (−0.15, 3.37)	−0.38 (−2.16, 1.38)
TG/HDL ratio	−0.03 (−4.16, 4.09)	1.29 (−2.50, 5.18)	−1.60 (−5.88, 2.68)

Linear regression models were used with adjustment of age, sex, energy and protein intakes

In the fully-adjusted Cox proportional hazards model, we observed an increasing trend in the risk of CHD events across dietary intake of total L-arginine (HR = 1.0, HR = 1.23, 95 % CI = 0.61 − 2.43, and HR = 1.39, 95 % CI = 0.69 − 2.66, P for trend = 0.07) (Table 4). When animal-derived L-arginine intakes were considered as exposure in the models, participants in the 2^nd^ tertile (1.45 − 1.78 g/d) had a significantly increased risk of CHD events compared to the participants in the 1^st^ tertile (<1.45 g/d) (HR = 1.90, 95 % CI = 1.03 − 3.58), while highest compared to the lowest intakes (<1.45 vs. ≥1.78 g/d) of animal-derived L-arginine was not related to the CHD risk (HR = 1.46, 95 % CI = 0.72 − 2.98). The risk of CHD had a decreasing trend across increasing plant-derived L-arginine intakes (HR = 1.0, HR = 0.91, 95 % CI = 0.51 − 1.62, HR = 0.72, 95 % CI = 0.39 − 1.32, P for trend = 0.03), however this negative association was not statistically significant. Hazard ratios (95 % CI) of CHD events per 1 SD increased intakes of total L-arginine, animal- and plant-derived L-arginine were 1.07 (0.63 − 1.81), 1.12 (0.72 − 1.73), and 0.90 (0.51 − 1.61), respectively.

**Table 4:** Hazard ratio (95 % CI) of coronary heart disease by tertiles of dietary intakes of L-arginine

	Dietary intakes of L-arginine (*n =* 1237)			
	1st tertile	2nd tertile	3rd tertile	1 SD increased intakes
Total L-arginine *(g/d)*	<3.25	3.25–4.42	≥4.42	
*Adjustment for age and sex*	Ref.	1.16 (0.59–2.29)	1.33 (0.69–2.59)	-
*Multivariate model 1*	Ref.	1.22 (0.61–2.41)	1.37 (0.68–2.60)	-
*Multivariate model 2*	Ref.	1.23 (0.61–2.43)	1.39 (0.69–2.66)	1.07 (0.63–1.81)
L-arginine from animal sources *(g/d)*	<1.45	1.45–1.78	≥1.87	
*Adjustment for age and sex*	Ref.	1.87 (1.01–3.48)	1.32 (0.65–2.66)	-
*Multivariate model 1*	Ref.	1.85 (0.99–3.45)	1.29 (0.64–2.60)	-
*Multivariate model 2*	Ref.	1.90 (1.03–3.58)	1.46 (0.72–2.98)	1.12 (0.72–1.73)
L-arginine from plant sources *(g/d)*	<1.85	1.85–2.20	≥2.20	
*Adjustment for age and sex*	Ref.	1.21 (0.62–2.35)	0.97 (0.50–1.89)	-
*Multivariate model 1*	Ref.	1.20 (0.61–2.38)	0.97 (0.48–1.92)	–
*Multivariate model 2*	Ref.	0.91 (0.51–1.62)	0.72 (0.39–1.32)	0.90 (0.51–1.61)

Cox proportional hazard regression models were used

Model 1: Additionally adjusted for BMI (kg/m^2^) and smoking status (yes/no)

Model 2: Additionally adjusted for hypertension (yes/no) and diabetes mellitus (yes/no)

## Discussion

In this prospective cohort study, during 4.7 years of follow-up of a sample of Iranian adults, some evidence regarding potential beneficial properties of plant-derived L-arginine along with adverse effects of animal-derived L-arginine intakes was observed in relation to cardiovascular risk factors and CHD events; total L-arginine intakes had no significant association with CHD risk or changes of serum lipids and blood pressure. A decreasing trend of CHD risk across increasing dietary intakes of plant-derived L-arginine, accompanied with a negative association with changes of systolic and diastolic blood pressure during the follow-up, may reveal the fact that plant sources of L-arginine have protective effects against development of cardiovascular diseases. In contrast, our findings indicated an increased risk of CHD and diastolic blood pressure levels in response to higher intakes of animal-derived L-arginine; the trend of HRs across animal-derived L-arginine seems not to be linear. These observations raised the hypothesis that different sources of dietary L-arginine may induce different cardiometabolic consequences.

Limited studies have examined the association of regular dietary intakes of L-arginine and the risk of cardio-metabolic disorders. L-arginine intake below the median range (3.8 g/d) was associated with higher levels of C reactive protein (CRP); highest level of L-arginine intake (>7.5 g/d) was also related to 30 % less likely to have a CRP above 3.0 mg/L [26]. Moreover, a lower prevalence of elevated SBP and LDL-C was also observed in subjects who consumed >7.5 g/d L-arginine [26]. Favorable effects of L-arginine supplementation, in doses ranging from 4 to 24 g/d, on blood pressure however have confirmed in a recent meta-analysis of 11 randomized, double-blind, placebo-controlled trials [9], hypotensive effect of regular dietary intakes of L-arginine have not been investigated in a population-based study.

Oomen et al., based on their findings from a population-based cohort study on elderly men, did not support the hypothesis that dietary L-arginine intake may lower the risk of CHD mortality; the relative risk for the medium and high tertiles compared to the lowest were 1.87 (95 % CI = 1.06 − 3.29) and 1.58 (95 % CI = 0.84 − 2.96), with a non-significant trend [14]. In this cohort study, a considerable decreasing trend of serum homosysteine levels were observed across increasing intakes of dietary L-arginine (13.9 *vs.* 17.8 μmol/L, *P* = 0.001, in L-arginine intakes ≥4.65 *vs.* 0 − 3.85 g/d, respectively); a lower prevalence of coronary artery disease and diabetes history was also observed in the highest compared to the lowest intakes of L-arginine [14]. Lack of stratified analysis for plant- and animal-derived L-arginine was one of the more important limitations for interpretation of these findings, and it is not clear that the association of L-arginine and CHD mortality to be affected from which sources of L-arginine.

In a 10-year follow-up of participants of Kuopio Ischemic Heart Disease Risk Factor Study, Venho et al. reported that total L-arginine as well as plant-derived L-arginine intake had no association with blood pressure or the risk of acute coronary events, whereas the highest quintile compared to the lowest quintile of animal-derived L-arginine had a marginally increased risk of acute coronary event after a 10 year follow-up (RR = 1.56, 95 % CI = 1.00 − 2.42) [15]; in this study, the portion of animal-derived L-arginine was higher than the plant-derived and there was an increasing trend in animal-derived L-arginine across quartiles of total intakes of L-arginine [15]. An interesting considerable point of Venho’s study and our findings was a non-linear trend of CHD risk across animal-derived L-arginine categorizations; Oomen et al. also reported similar trend for total L-arginine intake [14, 15]. Increased risk of CHD in the 2^nd^ tertile of total L-arginine and 5^th^ quintile of animal-derived L-arginine in Oomen and Venho cohorts was also comparable with our finding regarding positive association of animal-derived L-arginine with the risk of CHD.

Different findings on the association between plant- and animal-derived sources of L-arginine may be related to different metabolic response to plant- versus animal-derived protein [27]. It has been suggested that utilization of plant-derived L-arginine is better than animal-derived due to a higher ratio of lysine to L-arginine in animal proteins; lysine can compete with arginine for intracellular transport, and higher ratio of lysine to L-arginine may indirectly affect arginine metabolism [16]; therefore, it has been suggested that animal and plant sources of L-arginine induces different physiological consequence in the body [15].

In our study, a negative association of plant-derived L-arginine with CHD risk however, was not statistically significant. Inverse association with changes of both systolic and diastolic blood pressure may support speculations regarding cardioprotective effect of plant-derived L-arginine. Increased NO production have been reported in hypertension and cardiovascular disease [28–31]. Although current literature is rather confusing, it is suggested that overproduction of NO in these states is mainly due to increased inducible nitric oxide synthase (iNOS) activity [32]. Nitric oxide overproduction, as a compensatory response, has also been attributed to cytokines-dependent and insulin-dependent induction of iNOS, decreased endothelial nitric oxide synthase (eNOS) activity and reduced NO bioavailability [33–35]. Based on our observation, it can be speculated that pathogenic overproduction of NO, as a putative underlying mechanism involved in cardiovascular disease, may be prevented by a higher intake of plant-derived L-arginine.

In the current study, mean intake of dietary L-arginine was 4.0 ± 1.5 g/d and L-arginine intake was more from grains and meats and less from nuts and legumes. Mean intake of L-arginine in previous studies has been reported 4 − 6 g/d [26, 36]. Recommended dietary allowance has not yet been defined for L-arginine intake; soy protein, peanuts, walnuts and fish meats are rich sources while cereals and grains contain lower levels of L-arginine. Different dietary patterns between populations, therefore, may account for differences in mean intake of L-arginine. Mean arginine intake for the US adults is reported to be 4.40 g/day, with 25 % of people consuming < 2.6 g/d [36]. Median L-arginine intake in an adult population, participants of the National Health Nutrition and Examination Survey was also estimated to be 3.8 g/d. The highest level (90^th^ percentile) intake of L-arginine in our population (6.1 g/d) was also within the range of previous reports (4.5 − 7.5 g/d) [26].

The strengths of the current study were a population-based prospective setting, and use of a validated FFQ to assess regular dietary intake that provided an accurate estimation for dietary L-arginine intake. Lack of data on serum levels of L-arginine may be considered as an important limitation of this study.

## Conclusion

In conclusion, our findings suggested a potential protective effect of plant-derived L-arginine intake in regulation of blood pressure and prevention of CHD; moreover, higher intake of L-arginine from animal sources could be a dietary risk factor for development of HTN and cardiovascular risk factors.
